# Identification and validation of an immune-related gene prognostic signature for clear cell renal carcinoma

**DOI:** 10.3389/fimmu.2022.869297

**Published:** 2022-07-22

**Authors:** Shan Hua, Zhiwen Xie, Yongqing Zhang, Lei Wu, Fei Shi, Xingjie Wang, Shujie Xia, Shengli Dong, Juntao Jiang

**Affiliations:** ^1^ Department of Urology, Shanghai General Hospital, Shanghai Jiao Tong University School of Medicine, Shanghai, China; ^2^ Department of Urology, Shanghai General Hospital of Nanjing Medical University, Shanghai, China; ^3^ Nursing Department, Shanghai General Hospital, Shanghai Jiao Tong University School of Medicine, Shanghai, China

**Keywords:** clear cell renal carcinoma, immune microenvironment, immune-related genes, prognostic signature, immunotherapy

## Abstract

Clear Cell Renal Carcinoma (ccRCC) accounts for nearly 80% of renal carcinoma cases, and immunotherapy plays an important role in ccRCC therapy. However, the responses to immunotherapy and overall survival for ccRCC patients are still hard to predict. Here, we constructed an immune-related predictive signature using 19 genes based on TCGA datasets. We also analyzed its relationships between disease prognosis, infiltrating immune cells, immune subtypes, mutation load, immune dysfunction, immune escape, etc. We found that our signature can distinguish immune characteristics and predict immunotherapeutic response for ccRCC patients with better prognostic prediction value than other immune scores. The expression levels of prognostic genes were determined by RT-qPCR assay. This signature may help to predict overall survival and guide the treatment for patients with ccRCC.

## Introduction

Clear cell renal carcinoma (ccRCC) accounts for nearly 80% of renal carcinoma cases, and 76,080 new ccRCC cases and 13,000 ccRCC-related deaths were reported in the United States in 2020 (1, 2). Surgery is the most traditional treatment for ccRCC. However, with its innate high invasiveness and strong resistance to traditional therapy, such as radiotherapy and chemotherapy, advanced ccRCC is associated with high morbidity and mortality rates (3). Therefore, searching for new therapeutic targets and strategies and improving the prognosis of ccRCC are of high importance.

The prognosis of malignant tumours is intimately connected with pathological immune responses, which include antigen presentation, phagocytosis, and lymphocyte activation. Immune checkpoint inhibitors (ICIs), a type of cancer immunotherapy, are a revolutionary breakthrough in cancer treatment and have experienced significant advances in the last decade. ICIs targeting CTLA4 or PD-1 can effectively release T cells from suppression and restore antitumour immunity in the tumour microenvironment (TME) and have been applied in clinical practice (4). Although ICIs improve the prognosis for some patients, the overall clinical effects, including low immune-related adverse events and low response rates, are still unsatisfactory (5). However, the understanding of the TME of ccRCC is still limited, and we urgently require more robust biomarkers that can predict the prognosis and immune status and guide further treatment.

In this paper, we developed a prognostic signature for ccRCC to predict the prognosis after immunotherapy and traditional therapy. This paper mainly focused on immune-related genes in ccRCC transcriptomic data and constructed an immune-related gene prognostic signature with key immune-related genes, which were also related to the prognosis of ccRCC patients, identified in these data. The molecular and immune profiles of the signature were also characterized, and its prognostic value for immunotherapy patients was assessed and compared with tumour immune dysfunction and exclusion (TIDE) and the tumour inflammation signature (TIS). Our study indicated that our signature is a robust biomarker for predicting the prognosis of patients who receive conventional and immunotherapy.

## Materials and methods

### Acquisition the raw data

We retrieved the RNA sequencing (RNA-seq) profiles, clinical data, and gene mutation information from 72 normal and 539 clear cell renal carcinoma tissue in TCGA-KIRC datasets in The Cancer Genome Atlas (TCGA) (https://tcga-data.nci.nih.gov/tcga/). The RNA-seq and survival time for all samples in GSE29609 and GSE22541 were downloaded from the Gene Expression Omnibus (GEO) database (https://www.ncbi.nlm.nih.gov/geo/), and platform annotation file GPL1708 and GPL570 were also retrieved to annotate the probes. We obtained immune-related genes as comprehensive as possible in two online databases, the ImmPort (https://www.immport.org/shared/home) and InnateDB (https://www.innatedb.com/).

### Screening the differentially expressed immune-related genes

The RNA-seq profiles of all the 611 samples were combined to construct a mRNA matrix with Ensemble gene ID used for annotation. Then, Ensemble gene IDs were mapped to their corresponding gene symbols using human GTF file obtained from Ensembl (http://asia.ensembl.org). To identify the differentially expressed genes, the RNA-seq profiles obtained from TCGA were analyzed using limma R package with |log_2_FC| > 1.0, P < 0.05, and FDR < 0.05 as selection criteria, and the results were visualized using pheatmap R package. The DEIRGs were the intersection of these genes and immune-related genes obtained from ImmPort and Innate. The DEIGRs were then performed Gene Ontology (GO) and Kyoto Encyclopedia of Genes (KEGG) enrichment analysis using clusterProfiler, enrichplot, GOplot package of R to examine the enrichment of terms.

We performed Weighted correlation network analysis (WGCNA) to identify hub genes. The Pearson correlation coefficient between each pair of genes was calculated and used to construct a similarity matrix, which was transformed into an adjacency matrix with a signed network and a soft threshold of β = 3. Then, a topological matrix was constructed, and the topological overlap measure (TOM) was used to describe correlations between genes. We clustered these genes using 1-TOM, which represented the distance of genes, and identified seven modules using the dynamic pruning tree. The genes in the brown module, which were most significant between tumour and normal tissue, were considered hub genes and used to construct the coexpression network with a weight between two genes greater than 0.2. Gene expression was retrieved from the TCGA and GEO databases, and then, batch correction was completed. The best cut-off value for overall survival (OS) was calculated using “surv_cutpoint” function of the R package “survminer”, and the survival- and immune-related hub genes were used for the following study. The mutation of each survival-related gene in each sample was also analysed and visualized by the maftools package of R.

### Construction and validation of the signature

A total of 19 genes that significantly affect OS were identified and used to construct the ccRCC signature using multivariate Cox regression analysis. We calculated the risk score for each sample in the TCGA and GEO databases by multiplying the expression level of genes by their weight in the multivariate Cox model and adding them together. The Kaplan–Meier (K-M) survival curves of the TCGA and GEO cohorts were used to evaluate the prognostic power for constructing the signature. Univariate and multivariate Cox regression analyses were also performed to assess its independent prognostic value.

### Comprehensive analysis of molecular and immune characteristics and ICI therapy in different subgroups

The gene set enrichment analysis (GSEA) method, based on the HALLMARK and KEGG gene sets, was used with the clusterProfiler package of R to identify the signalling pathways in which DEIRGs were involved (P<0.05 and FDR<0.25). Then, the cBioPortal database (cBioPortal for Cancer Genomics) was used to download genetic alteration information and the quality and quantity of genetic mutations in two subgroups, which were divided by riskScore using the Maftools R package. Then, the expression matrix of 539 ccRCC samples was uploaded to the CIBERSORT database and iterated 1,000 times to determine the proportions of 22 types of immune cells. The proportions of immune cells and clinicopathological factors of patients were compared between the two subgroups, and a landscape map illustrated the results. To evaluate the immune and molecular functions of subgroups, ssGSEA of gene signatures was performed, and their scores were compared between subgroups. The prognostic value of our signature for patients receiving immunotherapy was also assessed by survival analysis. Additionally, the time-dependent ROC curve was obtained to calculate the AUC, and the prognostic value of our signature, TIDE, and TIS were also compared with the timeROC package of R.

### Cell culture and transfection

CcRCC cell-lines ACHN, 769-P, 786-O and normal cell line HK-2 were purchased from the Chinese Academy of Sciences Committee on Type Culture Collection Cell Bank (Shanghai, China). ACHN cells were cultured with Minimum Essential Medium (Biological Industries, CT, USA) supplemented with 10% FBS (Gibco, USA), and other cells were cultured in RPMI-1640 medium (Biological Industries, CT, USA) supplemented with 10% FBS (Gibco, USA). siRNA BMP1, siRNA VIM and siRNA negative control were purchased from Thermo Scientific (CA, USA), and transfected with Lipofectamine 2000 reagent (Invitrogen, CA, USA). All the siRNA primer sequences were listed in **Table S1**.

### RNA extraction and real-time quantitative polymerase chain reaction

Total RNAs of ccRCC cell-Line ACHN, 769-P, 786-O and normal cell line HK-2 were extracted using TRIzol (Novabio, China). RNA and PrimeScript RT kit (Novabio, China) were used to synthesize complementary DNA (cDNA). According to the manufacture’s protocol, real-time quantitative polymerase chain reaction (RT-qPCR) was performed with gene-specific primers to determine the relative expression of genes using SYBR green and was analyzed using ABI 7500 Real-Time PCR system (Applied Biosystem). All the genes’ primers were purchased from EnzyArtisan (Shanghai, China), and the primer sequences were listed in **Table S1**. All experiments were performed for three independent measures.

### Western blotting

The experimental proteins were extracted using the whole cell lysates (Beyotime, Shanghai, China) and their concentration were measured by BCATM Protein Assay Kit (Thermo Scientific, MA, USA). After dividing the proteins with SDS-PAGE, proteins were transferred to polyvinylidene difluoride (PVDF) membrane (Millipore, MA, USA), and blocked in 5% skim milk in 0.1% TBST at 4°C overnight. The proteins were probed with BMP1, VIM and GAPDH, after which they were incubated with secondary antibody. Proteins were visualized with an ECL chemiluminescence kit (Boster, Wuhan, China).

### CCK8 assay

To measure the cell proliferative capacity, 769-P cells were seeded in to 96-well plates for CCK-8 assay. After 24 and 48 hours, the cells in each well were incubated with 10 µl CCK8 reagent (Beyotime) for 1 hour, after which the absorbance at a wavelength of 450 nm was measured. Three independent experiments were performed for replication.

### Transwell invasion assay

To measure the cell invasive capacity, we conducted Transwell invasion assay. 769-P cells were suspended in 200 μl serum-free RPMI 1640 medium with 1% bovine serum albumin and were added to the upper compartments of a 24-well Matrigel invasion chamber containing polycarbonate filters with 8-mm pores and coated with Matrigel (BD Biosciences, CA, USA). The lower chamber was added with 1640 medium with 10% FBS. After incubation for 24 hours, the invaded cells were fixed with 4% methanol, then stained with 0.1% crystal violet and counted.

### Statistical analysis

We compared continuous variables between two groups using independent t test, categorical data using χ^2^ test, and the TIDE score between groups using Wilcoxon test. Besides, we performed univariate survival analysis using K-M survival analysis with log-rank test, and multivariate survival analysis using the Cox regression model.A two-side *P* < 0.05 was considered significant throughout this paper.

## Results

### Immune-relate hub genes

**Figure 1:**
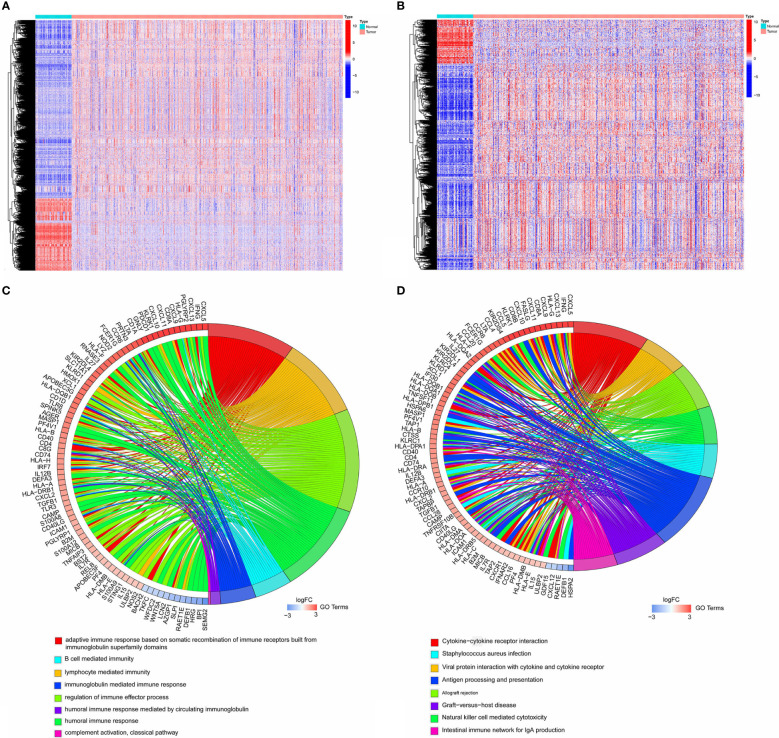
**(A)** Heatmap showing 1,909 genes downregulated and 4,903 upregulated in tumor samples by analyzing TCGA cohort. **(B)** Heatmap showing 166 immune-related genes downregulated and 780 immune-related genes upregulated in tumor samples. **(C, D)**. The top10 GO and KEGG terms by analyzing 946 DEIRGs.

A total of 6,812 genes were identified by the differential expression analysis for 539 tumors and 72 normal samples, including 1,909 genes downregulated and 4,903 upregulated (**Figure 1A**). The DEIRGs were the intersection of 6,812 genes and immune-related genes, including 166 genes downregulated and 780 genes upregulated (**Figure 1B**). The top GO term was adaptive immune response based on somatic recombination of immune receptors built from immunoglobulin superfamily domains, and the top KEGG term was cytokine-cytokine receptor interaction. The top 10 GO and KEGG terms were shown in **Figures 1C, D**.

**Figure 2:**
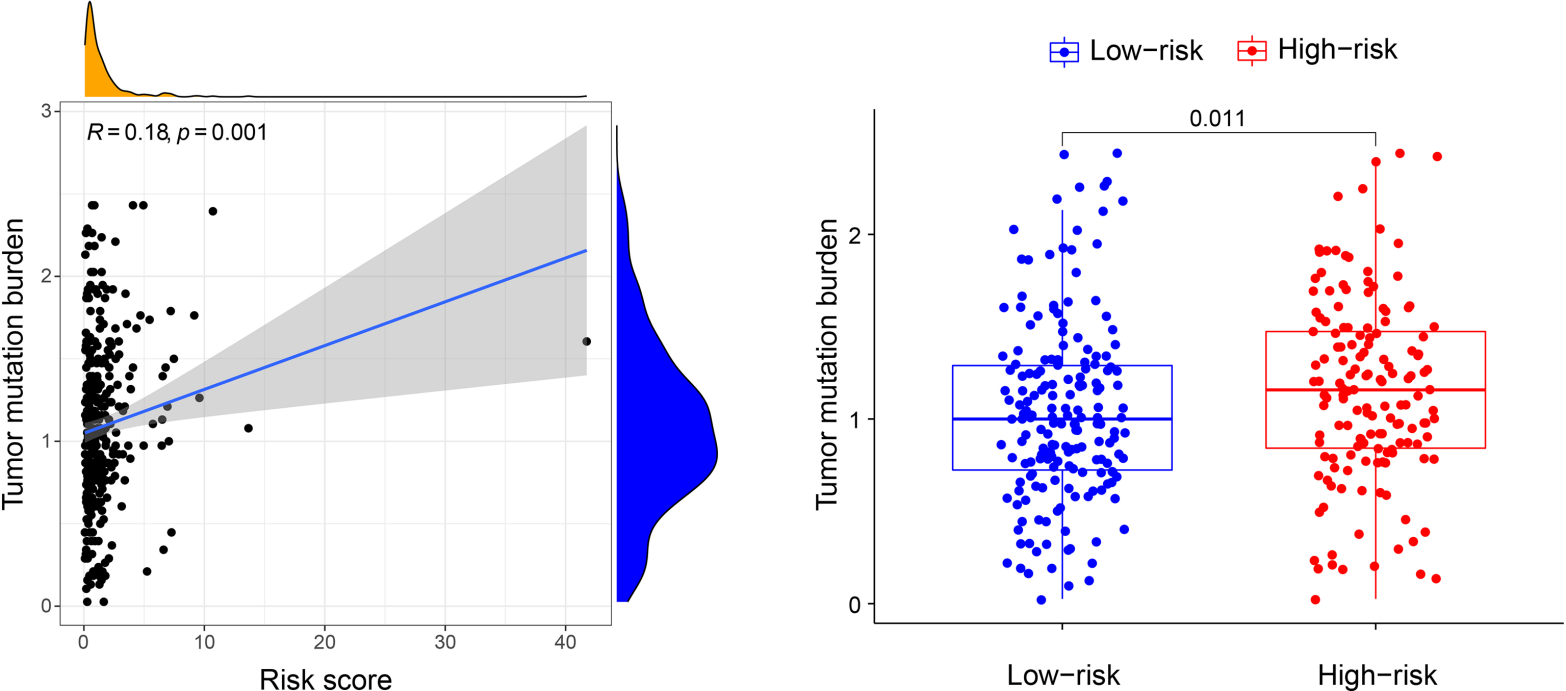
**(A)** Determination of the soft-thresholding power in the WGCNA analysis. The correlation coefficient was greater than 0.9, and the optimal soft-thresholding power was 3. **(B)** WGCNA of immune-related differentially expressed genes with a soft threshold β = 3. **(C)** Gene modules related to ccRCC obtained by WGCNA.

WGCNA was performed to identify the significant immune-related genes. The logarithm log(k) of the node in the coexpression network with connectivity K was negatively correlated with the logarithm log(P(k)) of the probability of the node. As shown in **Figure 2A**, the correlation coefficient was greater than 0.9, and the optimal soft-thresholding power was 3. All these DEIRGs were partitioned into seven modules based on soft-thresholding power and average linkage hierarchical clustering (**Figures 2B, C**). The Pierson correlation coefficient between sample features and modules was calculated in each module, and the brown module was the most highly correlated with ccRCC. A total of 137 genes in the brown module were used for further analysis. After merging these 137 genes with their clinical data in TCGA, 54 genes closely related to ccRCC patient OS were identified using K-M analysis (**Figure S1**). The frequency of mutations of each gene was obtained, and the rates of PDGFRA, PLAU, BMP1, FREM1, SEMA6D, KITLG, TEK, PRKCQ, and TRIM55 mutation were greater than 1% (**Figure S2**).

### Survival outcomes in different groups

We carried out multivariate Cox regression analysis to identify immune-related genes in order to predict prognosis for all ccRCC samples by the formula IRGP = TMSB4Y*(-0.36) + PLAU*(-0.217) + GNAI1*(0.399) + VIM*(0.299) + CDH1*(0.258) + VAV3*(-0.351) + SEMA3G*(-0.356) + SEMA6D*(-0.431) + BMP1*(0.627) + CHGA*(0.945) + IL11*(0.683) + TSLP*(0.787) + TACR1*(0.579) + TEK*(-0.327) + THRB*(-0.621) + PRKX*(-0.486) + TNIP1*(-0.606) + GATA4*(-1.142) + SREBF2*(0.705) (each gene name in this formula represents its expression level).

**Figure 3:**
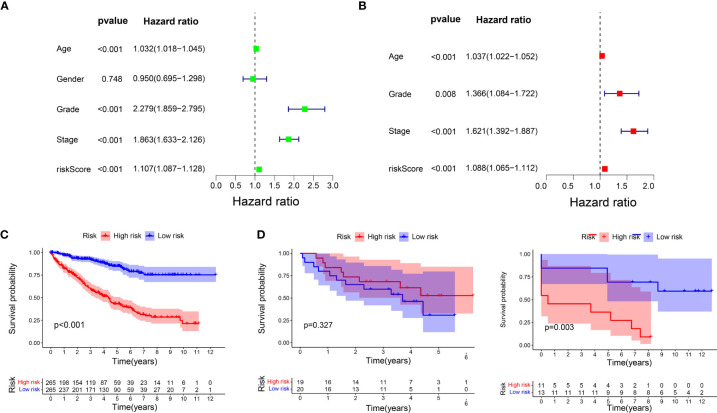
Prognostic analysis of the signature. **(A)** Univariate Cox regression analysis for the clinicopathological characteristics of 539 ccRCC samples in the TCGA cohort. **(B)** Multivariate Cox regression analysis for the clinicopathological characteristics of 539 ccRCC samples in the TCGA cohort. **(C)** K-M analysis of the signature in TCGA cohort. **(D)** K-M analysis of the signature in GSE29609 and GSE22541.

The results of univariate Cox regression analysis for the clinicopathological characteristics of 539 ccRCC samples in the TCGA cohort showed that age, grade, stage, and risk score were significantly related to the prognosis of ccRCC (**Figure 3A**). Multivariate Cox regression analysis showed the same conclusion as that of univariate Cox regression, which indicated that our signature could independently predict the prognosis of ccRCC patients (**Figure 3B**).

K-M analysis was carried out again to compare the OS of the two subgroups, which were divided using the median risk score as the cut-off value. The OS in the low-risk group was markedly longer than that in the high-risk group (*P* < 0.001, **Figure 3C**). We then used GSE29609 (n = 39) and GSE22541 (n = 24) to validate our signature. The patients’ OS for GSE29609 between the two groups was not significantly different (*P* = 0.327). For the GSE22541, whose ccRCC samples were all metastasis, the patients in high-risk group had worse prognosis (*P* = 0.003).

### Molecular characteristics of different subgroups

**Figure 4:**
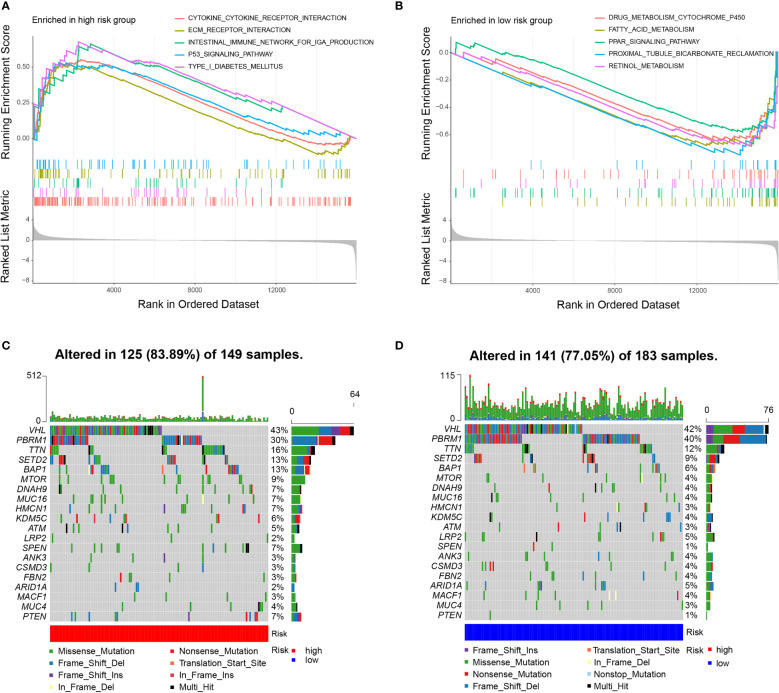
Molecular characteristics of subgroups. **(A)** Gene sets enriched in high-risk group (P < 0.05). **(B)** Gene sets enriched in low-risk group (*P* < 0.05). **(C, D)**. Mutated genes in the mutated ccRCC samples of two subgroups. Top 20 mutated genes (rows) are ordered by mutation rate; samples (columns) are arranged to emphasize mutual exclusivity among mutations.

We carried out GSEA to determine the enriched gene sets. IRDEGs in the high-risk group primarily enriched the interaction between small molecules and receptors (**Figure 4A**), and IRDEGs in the low-risk group primarily enriched the interaction between drug metabolism and acid metabolism-related pathways (**Figure 4B**).

Then, gene mutations in the two subgroups were analysed to investigate their further biological function in terms of immunological nature. The mutation frequencies of the top 20 most mutated genes are shown in **Figures 4C, D**. Generally, the mutation frequency in the high-risk group was much higher than that in the low-risk group. The mutation rates of TTN, SETD2, BAP1, MTOR, DNAH9, MUC16, HMCN1, SPEN, and PTEN were obviously higher in the high-risk group. Additionally, the mutation rates of LRP2, ANK3, CSMD3, FBN2, ARID1A, and MACF1 were slightly higher in the low-risk group than in the high-risk group. However, 40% of samples in the low-risk group had PBRM1 mutations compared to 30% in the high-risk group. In the two subgroups, missense mutations were the most common mutation type. The rates of mutation of VHL, PBRM1, and TTN were higher than 10% in both subgroups. To examine whether the risk scores and TMB were correlated, we performed regression analysis. The results showed that the risk score was significantly correlated with TMB (r = 0.18, p = 0.001), and the risk scores in high-risk group was higher than that in low-risk group (P = 0.011) (**Figure S3**).

### Immune characteristics of different subgroups

**Figure 5:**
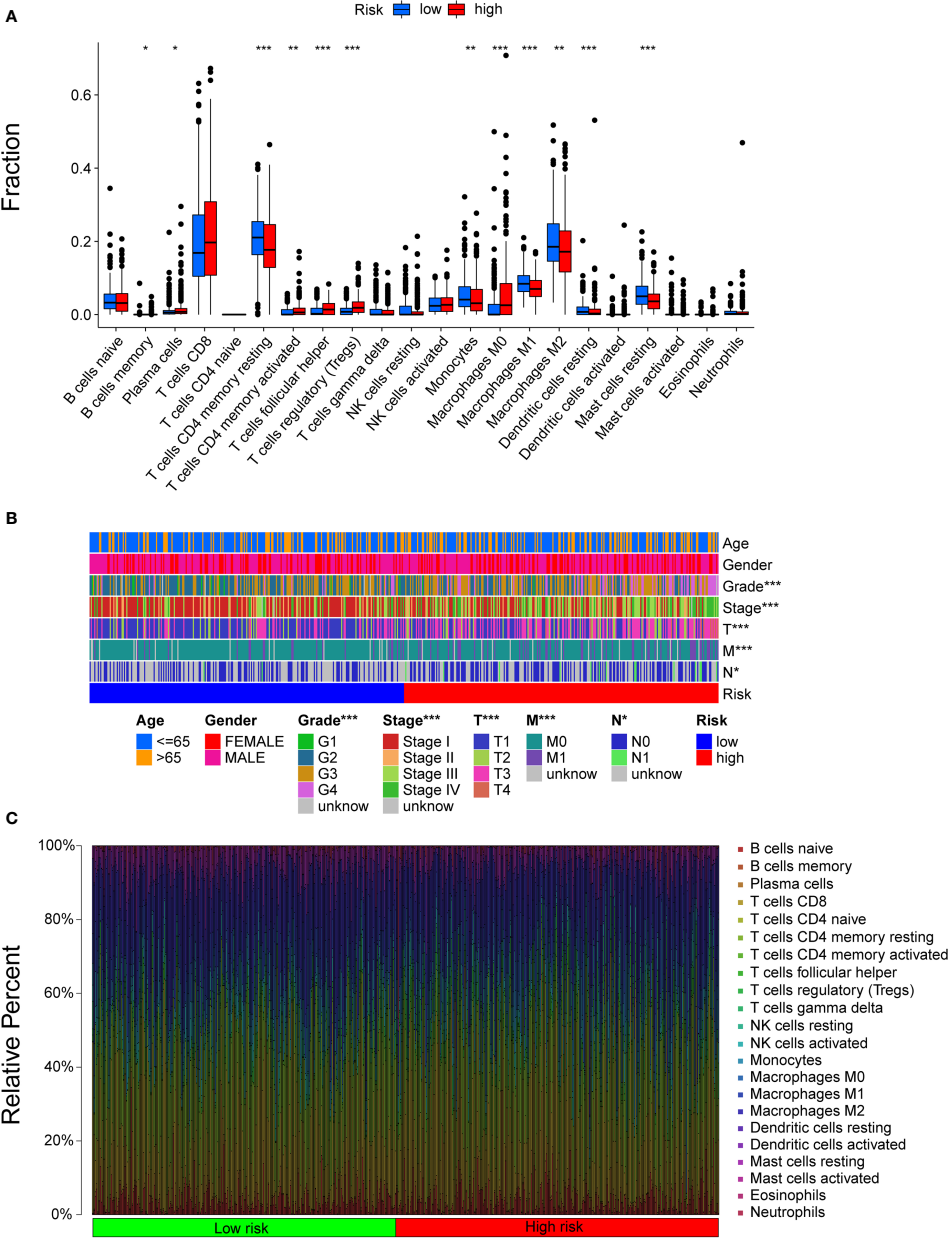
The landscape of the TME in ccRCC and the characteristics of different subgroups. **(A)** The distribution of TME cells in different subgroups. The thick lines are the median value, the bottom and top of the boxes are the 25th and 75th percentiles respectively. Significant statistical difference between subgroups were explores with the Wilcoxon test (ns, not significant; *P < 0.05; **P < 0.01; ***P < 0.001). **(B)** The clinicopathologic characteristics of different subgroups. **(C)** The distribution of cells in the TME of 530 patients.

All samples were divided into two groups by riskScores, and we used the Wilcoxon test to explore the difference in the distribution of immune cells between the two subgroups. The results indicated that resting memory CD4 T cells, monocytes, M1 macrophages, M2 macrophages, resting dendritic cells, and resting mast cells were more related to low risk scores, while plasma cells, activated memory CD4 T cells, regulatory T helper cells, gamma delta T cells, and M0 macrophages were more related to high risk scores (**Figure 5A**). **Figure 5B** shows that there were significant differences between the two subgroups in terms of grade, stage, and TNM stage. The distribution of cells in the TME of 530 patients in the TCGA cohort is shown in **Figure 5C**.

Then, some gene signatures were applied to identify the immune and molecular functions between the two subgroups. Patients with higher risk scores received higher scores of immune functions, including APC costimulation, DCs, CD8+ T cells, checkpoints, cytolytic activity, inflammation promotion, macrophages, CCR, parainflammation, T cell coinhibition and stimulation, T helper cells, Tfhs, Th1/2 cells, regulatory T cells (Tregs), and tumour infiltrating lymphocytes (TILs) (**Figure S4**).

K-M analysis indicated that immune functions were correlated with prognosis. We found that patients with high scores of resting DCs, naïve B cells, M2 macrophages, resting masting cells, monocytes, and resting CD4 memory cells had a worse outcome, while patients with higher scores of M0 macrophages, masting cells, plasma cells, activated memory CD4 T cells, follicular helper T cells, and Tregs had a better outcome (**Figure S5**). Thus, the robust predictive value of our signature might rely on better immune control.

### Relationship between subgroups and other immune clinical subtypes

**Figure 6:**
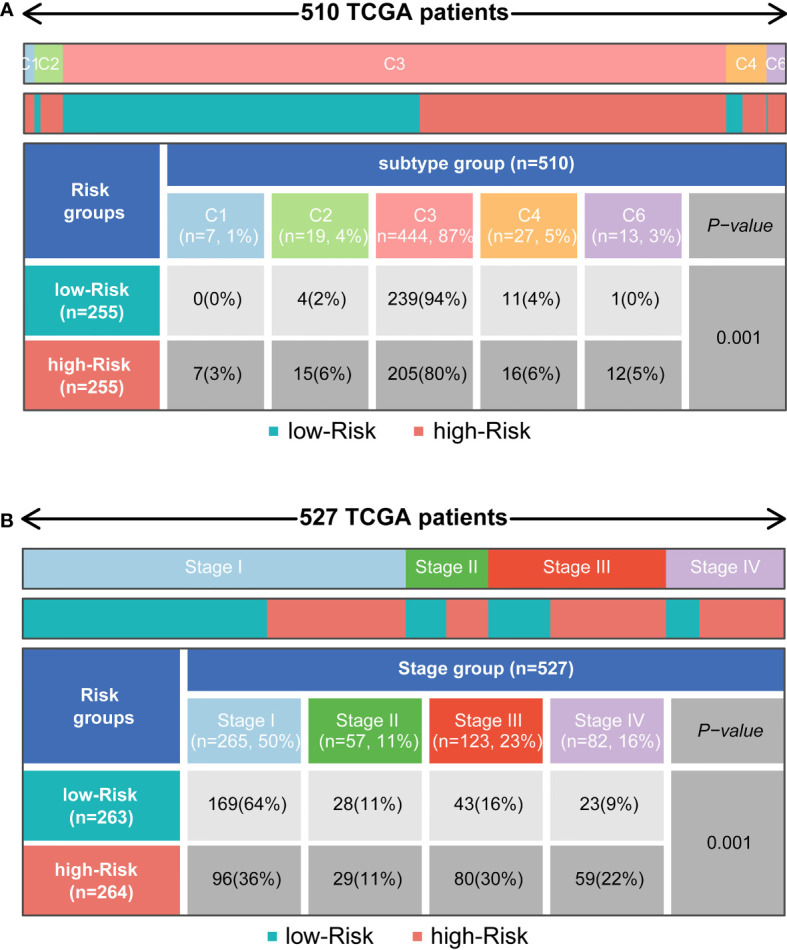
Distribution of immune subtypes and the clinicopathologic characteristics in different subgroups. **(A)** Heatmap and table showing the distribution of tumour immune subtypes (C1, C2, C3, C4, C5, C6) between subgroups. **(B)** Heatmap and table showing the distribution of clinicopathologic characteristics between subgroups.

To determine the immunophenotype, the cophenetic correlation coefficients were calculated to identify the best k value, and k = 6 was selected after comprehensive consideration (named C1, C2, C3, C4, C5, and C6). We found that most of the patients represented by the 510 TCGA samples were in the C3 subtype, and most of these patients belonged to the low-risk group. However, patients in other subtypes were more likely to be in the high-risk group (P < 0.001) (**Figure 6A**). For patients with different ccRCC stages, the tumour stage increased significantly with increasing risk scores (P < 0.001) (**Figure 6B**).

### The cenefit of ICI therapy in different subgroups

**Figure 7:**
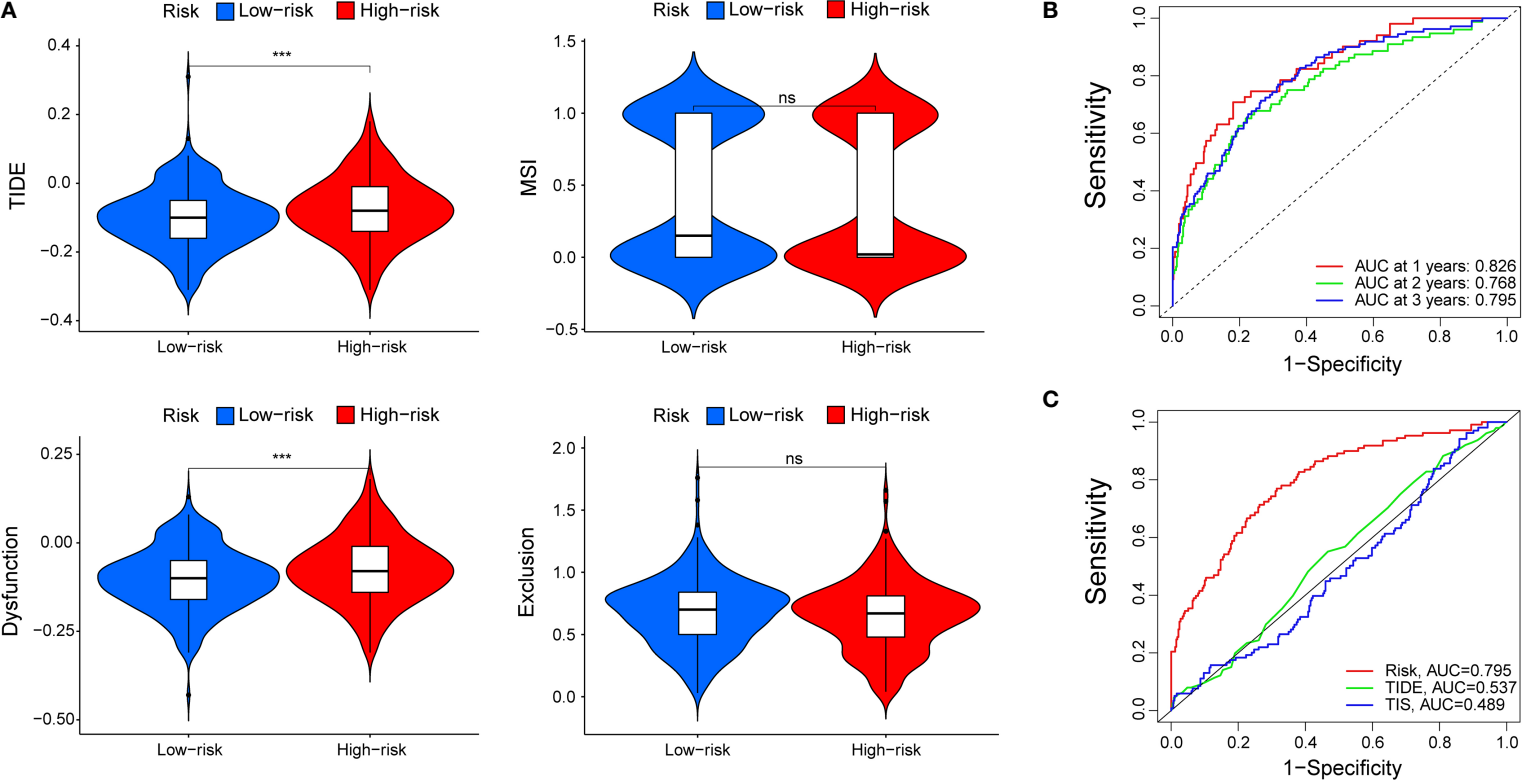
The prognostic value of the signature for patients with ccRCC. **(A)** TIDE, MSI, and T-cell dysfunction and exclusion score in different subgroups. The scores between the two subgroups were compared by the Wilcoxon test (ns, not significant; ***P < 0.001). **(B)** ROC analysis of our signature on OS at 1-, 2-, and 3-years follow-up. **(C)** ROC analysis of our signature, TIS, and TIDE on overall survival.

TIDE modeled tumour immune escape in the context of different cytotoxic T lymphocyte levels, and higher TIDE scores indicated a higher likelihood of immune evasion. As shown in **Figure 7A**, the patients with higher risk scores had higher TIDE scores than those with lower risk scores, which meant that patients with lower risk scores were more sensitive to ICI therapy than patients with higher risk scores. In other words, patients with lower TIDE scores probably had a better prognosis than those in the high-risk group who had high TIDE scores. In addition, we found that patients with higher risk scores were more likely to be affected by T cell dysfunction, while there was no significant difference in microsatellite instability (MSI) score or T cell exclusion between the two subgroups. Moreover, the AUCs of our signature at 1, 2, and 3 years were 0.826, 0.768, and 0.795, respectively, which meant that the signature had great predictive performance (**Figure 7B**). Compared with TIDE and TIS, the 3-year AUCs of our signature displayed a much higher performance (TIDE, AUC = 0.537; TIS, AUC = 0.489) (**Figure 7C**).

### Prognostic genes verified by RT-qPCR

**Figure 8:**
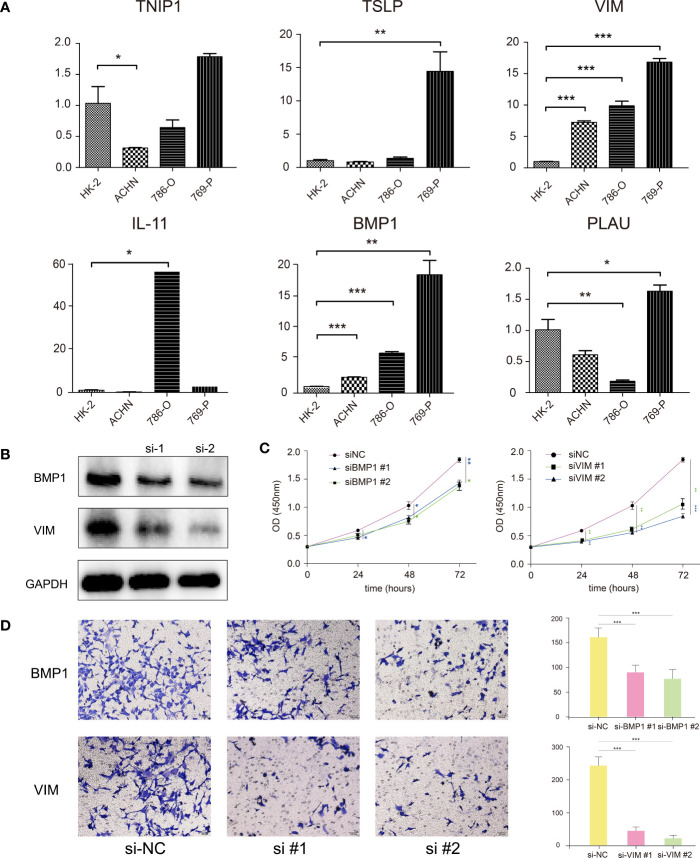
**(A)** RT-qPCR confirmed the difference of the prognostic gene expression in normal renal and renal cell carcinomas. **(B)** Western blotting showed that the BMP1 and VIM genes were effectively suppressed after siRNA transfection. **(C)** CCK-8 assay suggested that the proliferation ability of 769-P cells was reduced with BMP1 and VIM knockdown. **(D)** Transwell assay showed that the invasion ability of 769-P cells was reduced with BMP1 and VIM knockdown. *P < 0.05, **P < 0.01, ***P < 0.001.

To validate the different expression of our prognostic genes in this signature, RT-qPCR was performed to analyze the mRNA expression in ccRCC cell lines and normal cell line. BMP1, IL-11, TSLP, and VIM were significantly upregulated in certain or all ccRCC cell lines, TNIP1 was downregulated in ACHN cells, and PLAU had different expression level in different cell lines (**Figure 8A**). The RT-qPCR results for other genes with significant different express levels among ACHN, 786-O, and 769-P, or their express levels were too low in certain cell lines were shown in **Figure S6**.

### BMP1 and VIM genes affected the proliferation and invasion of 769-P cells

For verification, we explored the functions of two genes, BMP1 and VIM, significantly highly expressed in all three ccRCC cells, which had been verified in qRT-PCR. After siRNA transfection, the western blot analysis showed siRNA effectively represses the target genes in 769-P cells (**Figure 8B**). The CCK-8 assay showed the proliferation ability of 769-P cells with BMP1 or VIM knockdown was significantly reduced compared with cells in negative control group (**Figure 8C**). The Transwell assay showed that the invasion ability of 769-P cells with BMP1 and VIM knockdown was significantly suppressed (**Figure 8D**). All the results demonstrated that the candidate genes BMP1 and VIM were risk factors for ccRCC, which was in consistence with our signature.

## Discussion

Immunotherapies in the form of ICIs that target coinhibitory immune checkpoints to regulate the immune response have been widely used and have brought ccRCC therapy to a new era. However, in many cases, patients fail to respond or suffer from secondary drug resistance after a short effective course and cancer progression (6). There is still no well-accepted signature to predict patients’ response to immunotherapy for ccRCC. Thus, it is of interest to identify a robust biomarker for ccRCC patients to predict immunotherapy sensitivity.

The TME of tumours exhibits different responses to ICIs due to polygenic effects, and WGCNA is an effective approach to identify key immune-related genes. Combined with clinical data from TCGA, 19 key genes that affect OS were identified and used to construct the risk model. Our risk model was an independent prognostic immune-related indictor for ccRCC, with higher risk scores representing worse prognosis in TCGA. However, the result of K-M analysis of GSE29609 was not statistically significant (**Figure 3D**). This result most likely occurred to do the small sample size, which may result in bias and statistical error. For metastatic ccRCC patients in the GSE22541, which only contained metastatic samples, patients in high-risk group had significant shorter overall survival. It may suggest that our signature is more applicable for metastatic ccRCC patients. However, we should note that the sample size was also too small, the results may not accurate enough. To demonstrate the accuracy of the risk model, prospective studies with large samples should be performed in the future.

This risk model included nineteen genes. Among these genes, several familiar genes play a critical role in the regulation of cancer. Bone morphogenetic protein 1 (BMP1), a secreted metalloprotease, initially cleaves and releases the TGF-β complex from the matrix. BMP1 is involved in the TFG-β and BMP signalling pathways, and it has been reported that BMP1 expression upregulation is involved in gastric, lung, and colon cancer progression (7–9). A recent study pointed out that high expression of BMP1 might cause poor outcomes, and knockdown of BMP1 could suppress ccRCC progression (10). Fragments of chromogranin A (CHGA), an acidic glycoprotein frequently used as a prognostic factor for many neuroendocrine tumours, can affect critical components of the TME, such as fibroblasts and endothelial cells, as well as tumour progression and immunotherapy in patients (11, 12). Interleukin 11 (IL-11) is commonly thought to exert pro-oncogenic effects through the JAK-STAT3 signalling pathway, whose overactivation could suppress the immune response by the differentiation of macrophages, DCs, and polymorphonuclear leukocytes (13, 14). Thymic Stromal Lymphopoietin (TSLP) regulates barrier immunity in tumors by activating T cells and DCs to promote TME favorable. A study indicated that CAFs in GATA3+ breast tumours could produce TSLP+ DCs, which exist in tumour-draining lymph nodes but not in nondraining lymph nodes. In addition, TSLP plays a role in the mechanisms underlying the TME of cervical cancer, gastric, and ovarian cancer (15–18). Substantial evidence indicates that the mutation of tumour necrosis factor α-induced protein 3-interacting protein 1 (TNIP1) may increase the risk of SLE and LN by regulating the canonical NF-κB pathway (19). Additionally, miR-210-3p persistently activates the NF-κB pathway by targeting its negative regulator TNIP1 in prostate cancer and therefore promotes EMT, invasion, migration, and bone metastasis (20). The expression of GATA Binding Protein 4 (GATA4) showed the most significant negative correlation with risk scores in our signature. GATA4 probably suppresses lung cancer through the TGF-β2/Wnt7B signalling pathway, and GATA deficiency blunted the therapeutic effect of MEK1/2 inhibition in a mouse model (21). Sterol regulatory element binding transcription factor 2 (SREBF2), an important regulator of cholesterol biosynthesis, has been proven to be significantly differentially expressed in the prostate cancer DU145 cell line, and its expression, and thus its pro-oncogenic role, might be regulated by miR-28-5p (22).

Further study focusing on the immunological nature of our signature was necessary; therefore, we explored gene mutations in different subgroups. VHL and PBRM1 had similar mutation frequencies and the two highest mutation frequencies in patients with ccRCC, and this result was concordant with previous reports that PBRM1 inactivation generally coincided with mutation of VHL because they are found close together on chromosome arm 3p. Focal or whole-arm deletions generally affect VHL and PBRM1 simultaneously. Interestingly, conventional wisdom suggests that PBRM1 is a tumour suppressor gene, and its silencing would cause proliferation, migration, and colony formation in ccRCC. PBRM1 mutation affects p53-dependent chromatin regulation and triggers immune escape mediated by p-53 in ccRCC tumours (23). Additionally, the inactivation of PBRM1 was associated with a less immunogenic TME, resulting in decreased immune infiltration and poor response to ICIs, especially in ccRCC (24–26). Interestingly, the mutations of PBRM1 in our signature were in contrast to the traditional view that mutations were more common in patients with lower risk scores (40% vs. 30%). A recent study reported that the role of PBRM1 in ccRCC was context-dependent. The mutation of PBRM1 in ccRCC 786-O cells, in which HIF1α was not fully expressed, suppressed survival and proliferation by decreasing the expression of a key pro-oncogenic factor, HIF2α, which results in the accumulation of hypoxia-inducible factors that drive dysregulated angiogenesis (27, 28). It is worth noting that the subgroups in our signature were divided by the OS of patients in TCGA regardless of the difference in cell lines, and the signature was constructed from an overall perspective. In the future, it will be valuable to reveal different mechanisms by which mutations in PBRM1 exert diverse effects on cancer progression and the TME among ccRCC cell lines. For TTN, which exhibited the third highest mutation rate in both subgroups and whose mutation was more common in patients with high risk scores, it has been reported that its mutation contributed to TMB and affected the cell cycle, metabolism, DNA repair, immune cell infiltration, immune checkpoint expression, and thus the prognosis in some solid tumours (29). Additionally, sufficient studies have suggested that SETD2, BAP1, mTOR, MUC16, HMCN1, KDM5C, ARID1A, and PTEN are intimately tied to prognosis and immune response in cancers (30–35).

The relationship between risk scores calculated using the signature and TMB, which is a potential biomarker that predicts the treatment effect of ICIs in many cancers, including ccRCC, was determined (36). Here, the risk scores showed a strong correlation with TMB, which indicated that our signature could be used to predict immunotherapy prognosis to some extent. The landscape of the TME revealed that the two subgroups were composed of different immune cells, which could help to identify or improve therapeutic approaches for the enhancement of immune responses. Activated memory CD4 T cells, Tfhs, Tregs and M0 cells were mainly enriched in the high-risk group, while M1 cells, M2 cells and DCs were more enriched in the low-risk group. Tregs maintain tumour immune exclusion through various mechanisms, including affecting immune and nonimmune cells inside or outside of the tumours. The immunosuppressive activity of Tregs is considered a main barrier to effective antitumour immunity in ccRCC (37). M1 macrophages play an important role in producing inflammatory cytokines and evoking the immune response (38). Our immune landscape might provide a theoretical basis for further study of ccRCC treatments.

Six stable tumour immune subtypes, which were identified by prognostic and genetic and immune modulatory alterations, were identified in 2018 and have been reproduced more than one thousand times to date. This typing method has been widely accepted and helps us to understand the tumour immune environment (39). According to the immune subtype, more patients with low risk scores were in the C3 subtype, whereas the other immune subgroups were correlated with high risk scores. C3 is characterized by elevated Th17 and Th1 gene expression, low to moderate tumour cell proliferation and lower levels of aneuploidy and somatic copy number alterations. Of note, C3 is enriched in PBRM1 mutations, which is consistent with our study, and PBRM1 mutations generally respond well to immunotherapy. Additionally, C3 always disrupts the TGF-β pathway, which is related to more abundant M1 macrophages and lower proportions of helper T cells and M0 macrophages. C1 and C2 conferred poor prognosis despite a large number of infiltrating immune cells. Enhancement of T cell activity, nevertheless, could improve the prognosis of these patients. The C4 and C6 subtypes exhibit lower lymphocyte infiltration and higher M2 macrophage infiltration, which is consistent with the immunosuppressive TME and therefore correlates with poor prognosis (39). Our study indicated that patients in the low-risk group probably exhibit active immunity with better outcomes. In addition, the validation of the signature with clinical relevance implied that higher risk scores were correlated with higher tumour stage, which was in accordance with our results.

Moreover, we also found that our signature could reflect the diverse immune benefits of ICI therapy as determined with TIDE, which has been widely used to predict the outcome of patients with cancer treated with ICIs more accurately than other biomarkers, such as TMB and PD-L1 (40). The results suggested that patients with higher risk scores had more cytotoxic T lymphocyte infiltration and higher TIDE and T cell dysfunction scores than those with lower risk scores, which indicates poor outcomes of ICI treatment in patients with high risk scores due to immune evasion. Another biomarker that predicts response to immunotherapy is TIS, which is an 18-gene signature measuring a pre-existing but suppressed immune response in tumours. The expression pattern of these genes has been proven to be conserved across tumour types (41). The expression pattern of these genes has been proven to be conserved across tumour types. Both TIDE and TIS, however, only consider to T cells, and it is difficult to reflect the overall status of the TME and survival time. In this study, the predictive value of our signature was reliable and much better than that of TIDE and TIS.

## Conclusion

We constructed a very promising and comprehensive immune-related gene signature to predict the prognosis of ccRCC. This signature may help to distinguish immune characteristics and predict diseases outcomes. Meanwhile, it might play an important role in prediction of immunotherapeutic response.

## Data availability statement

The original contributions presented in the study are included in the article/**Supplementary Material**. Further inquiries can be directed to the corresponding authors.

## Author contributions

SH designed the study, ran the R and Perl codes, and experimental verification. ZX and YZ checked and corrected the R and Perl codes, collected and processed raw data; LW wrote the manuscript; XW and FS performed the statistical analysis and revised the manuscript; SX revised the manuscript and provide methodological guidance; SD and JJ improved the study design, funded and supervised the study. All authors contributed to the article and approved the submitted version.

## Funding

This work was supported by The National Natural Science Foundation of China (No.81771564) and Zunyi Municipal Science and technology Bureau [NO. 2018(192)].

## Conflict of interest

The authors declare that the research was conducted in the absence of any commercial or financial relationships that could be construed as a potential conflict of interest.

## Publisher’s note

All claims expressed in this article are solely those of the authors and do not necessarily represent those of their affiliated organizations, or those of the publisher, the editors and the reviewers. Any product that may be evaluated in this article, or claim that may be made by its manufacturer, is not guaranteed or endorsed by the publisher.
