# Supplementary figures and images for: Identification and validation of an immune-related gene prognostic signature for clear cell renal carcinoma
Source: Front Immunol. 2022 Jul 22;13:869297. doi: 10.3389/fimmu.2022.869297 (PMC9352939; doi:10.3389/fimmu.2022.869297)

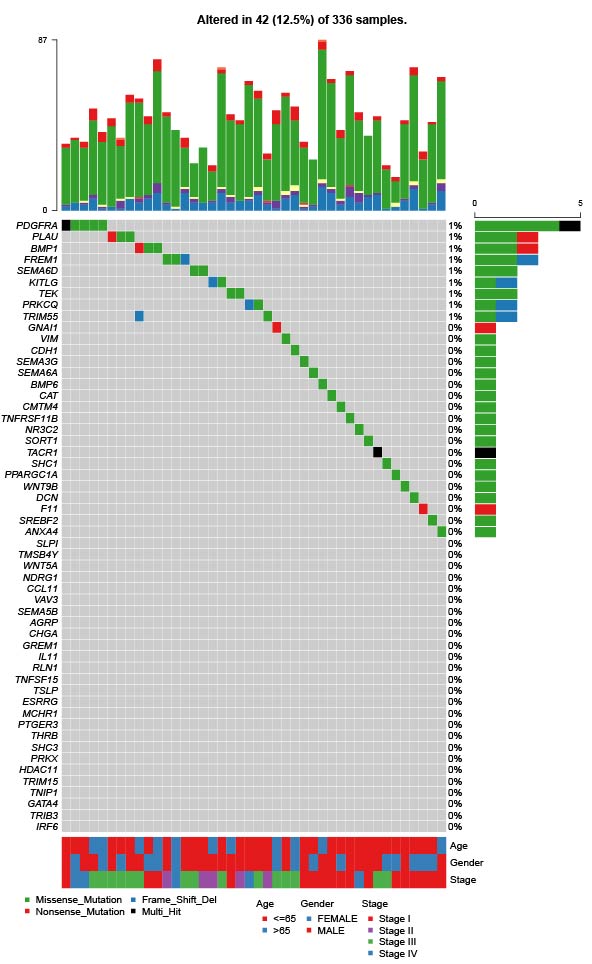

Supplement: Supplementary file 2 [file Image_2.jpeg]

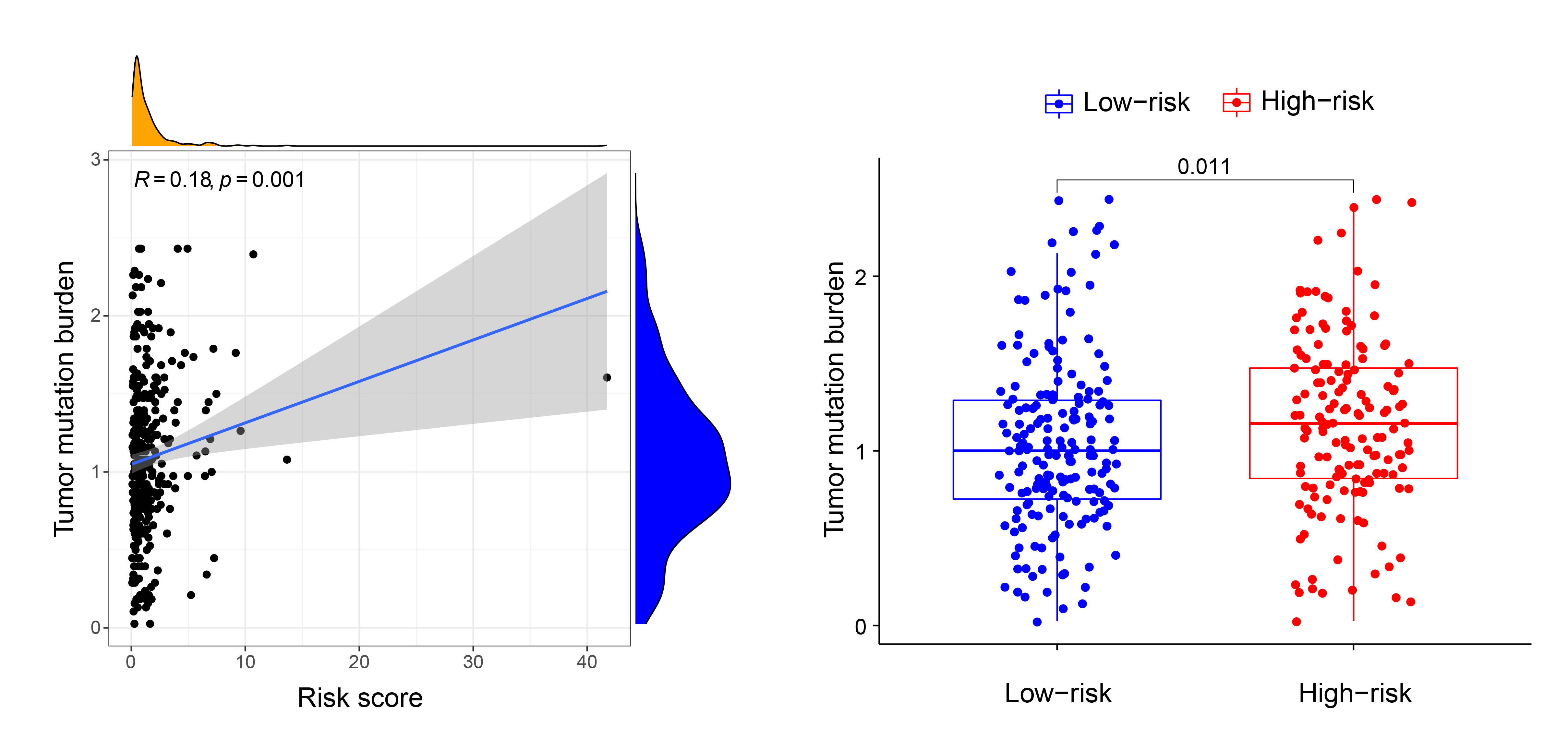

Supplement: Supplementary file 3 [file Image_3.jpg]

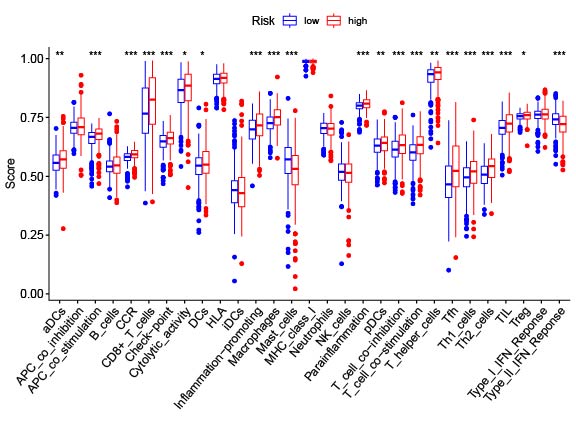

Supplement: Supplementary file 4 [file Image_4.jpeg]

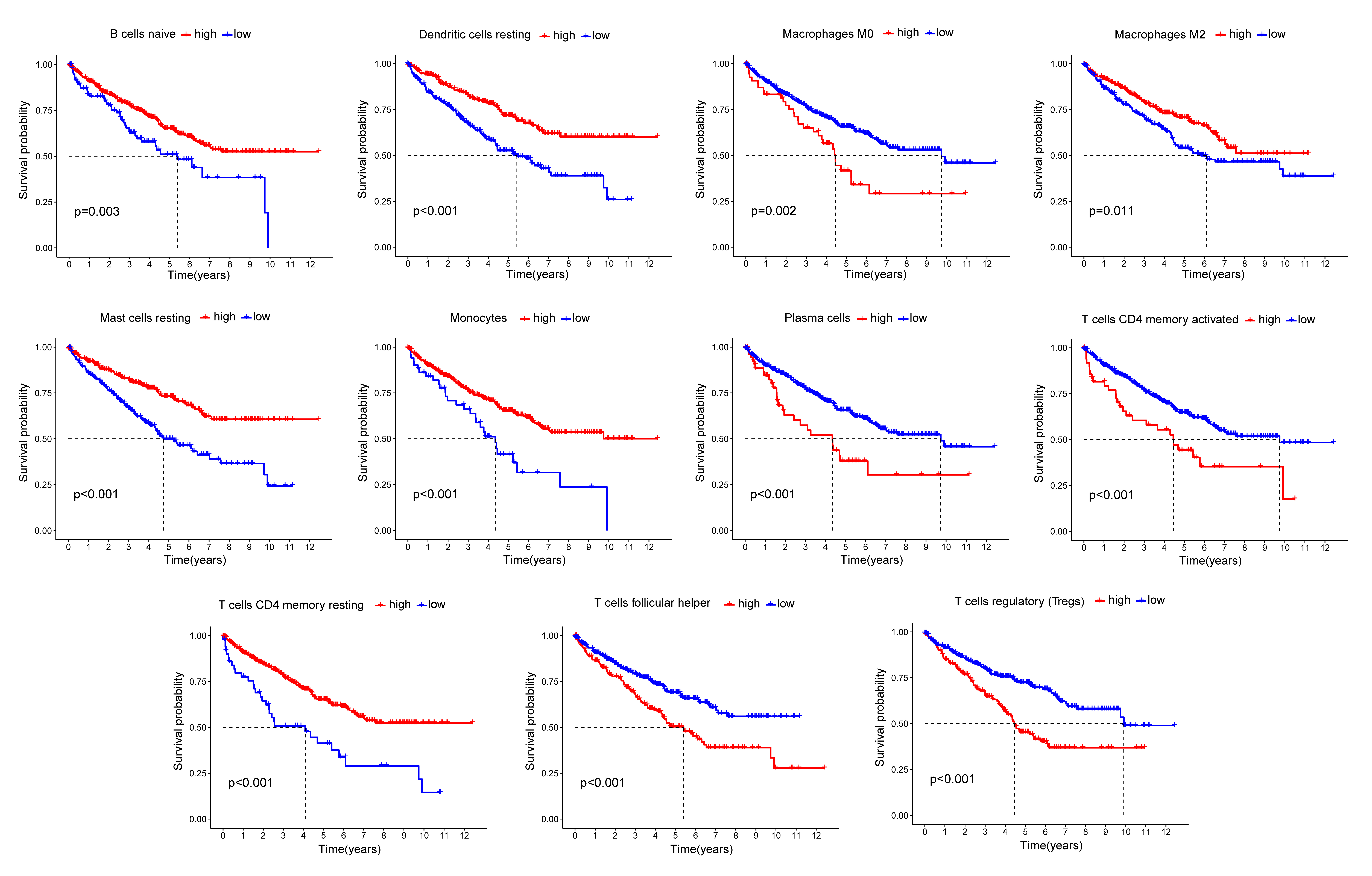

Supplement: Supplementary file 5 [file Image_5.jpeg]

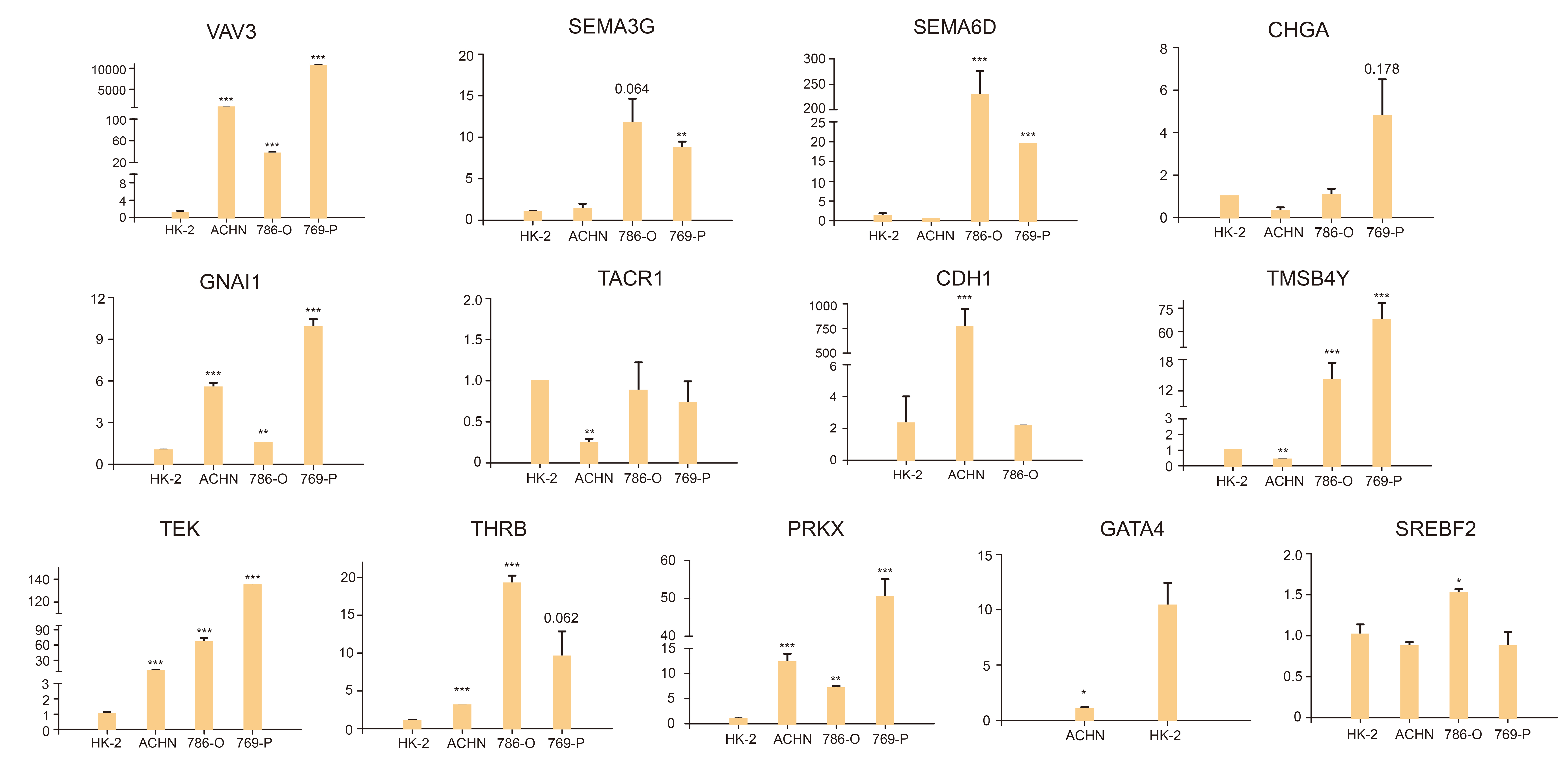

Supplement: Supplementary file 6 [file Image_6.jpeg]
