# Supplementary material for: Identification and validation of an immune-related gene prognostic signature for clear cell renal carcinoma
Source: Front Immunol. 2022 Jul 22;13:869297. doi: 10.3389/fimmu.2022.869297 (PMC9352939; doi:10.3389/fimmu.2022.869297)
Supplement: Supplementary file 7 [file Table_1.docx]

Table S1

| Control siRNA |  |
| --- | --- |
| sense: | UUCUCCGAACGUGUCACGUTT |
| antisense: | ACGUGACACGUUCGGAGAATT |
| BMP1 siRNA #1 |  |
| sense: | GCUAUAUUGUGUUCACCUATT |
| antisense: | UAGGUGAACACAAUAUAGCTT |
| BMP1 siRNA #2 |  |
| sense: | GCACUGACGAGGACAGCUATT |
| antisense: | UAGCUGUCCUCGUCAGUGCTT |
| VIM siRNA #1 |  |
| sense: | GGCUUUAGCGAGUUAUUAATT |
| antisense: | UUAAUAACUCGCUAAAGCCTT |
| VIM siRNA #2 |  |
| sense: | GCUUUAGCGAGUUAUUAAATT |
| antisense: | UUUAAUAACUCGCUAAAGCTT |
| CHGA |  |
| Forwards | GGTTCTTGAGAACCAGAGCAGC |
| Reverse | GCTTCACCACTTTTCTCTGCCTC |
| SEMA6D |  |
| Forwards | GCATCTCGTGACCCGTATTGTG |
| Reverse | CCTAGATGAGCTGTGTTGCCGA |
| SEMA3G |  |
| Forwards | GCTCAAAGTCATCGCTCTCCAG |
| Reverse | CTCCATTTCGGTGATAGGTGTTG |
| TMSB4Y |  |
| Forwards | ACCTGGTATGGCTGAGATCGAG |
| Reverse | GCTTGCCTCTCCTGTTCGATAG |
| PLAU |  |
| Forwards | GGCTTAACTCCAACACGCAAGG |
| Reverse | CCTCCTTGGAACGGATCTTCAG |
| GNAI1 |  |
| Forwards | AGCACTGAGTGACTACGACCTG |
| Reverse | GGATGTATCTGTAAACCACTTGTTG |
| VIM |  |
| Forwards | AGGCAAAGCAGGAGTCCACTGA |
| Reverse | ATCTGGCGTTCCAGGGACTCAT |
| CDH1 |  |
| Forwards | CCCAATACATCTCCCTTCACAG |
| Reverse | CCACCTCTAAGGCCATCTTTG |
| VAV3 |  |
| Forwards | AAATTTAGCATCTGGAGAGGTTGG |
| Reverse | GAATAATCTACTGGTTTGGGCACAC |
| BMP1 |  |
| Forwards | CCAATGGCTACTCTGCTCACATG |
| Reverse | AAGCCATCTCGGACCTCCACAT |
| IL11 |  |
| Forwards | GGACCACAACCTGGATTCCCTG |
| Reverse | AGTAGGTCCGCTCGCAGCCTT |
| TSLP |  |
| Forwards | TATCTGGTGCCCAGGCTATTCG |
| Reverse | TGAAGCGACGCCACAATCCTTG |
| TACR1 |  |
| Forwards | GCCTGTTCTACTGCAAGTTCCAC |
| Reverse | CACAGATGACCACTTTGGTGGC |
| TEK |  |
| Forwards | GGTCAAGCAACCCAGCCTTTTC |
| Reverse | CAGGTCATTCCAGCAGAGCCAA |
| THRB |  |
| Forwards | CGAGAACACGACTGGAAGCTAG |
| Reverse | CCAAGTGGTCTGGATGAGATGTG |
| PRKX |  |
| Forwards | TCGTGGTTGACAGAACAAGGCG |
| Reverse | CACCAGCTATCTTGGGCACGAT |
| TNIP1 |  |
| Forwards | GAGTTCAACCGACTGGCATCCA |
| Reverse | TTCAGAGCCTCGTTCTCCTTCC |
| GATA4 |  |
| Forwards | GCGGTGCTTCCAGCAACTCCA |
| Reverse | GACATCGCACTGACTGAGAACG |
| SREBF2 |  |
| Forwards | CTCCATTGACTCTGAGCCAGGA |
| Reverse | GAATCCGTGAGCGGTCTACCAT |
